# Understanding the role of GPs’ gut feelings in diagnosing cancer in primary care: a systematic review and meta-analysis of existing evidence

**DOI:** 10.3399/bjgp20X712301

**Published:** 2020-08-25

**Authors:** Claire Friedemann Smith, Sarah Drew, Sue Ziebland, Brian D Nicholson

**Affiliations:** Nuffield Department of Primary Care Health Sciences, University of Oxford, Oxford.; London School of Economics and Political Science, London.; Nuffield Department of Primary Care Health Sciences, University of Oxford, Oxford.; Nuffield Department of Primary Care Health Sciences, University of Oxford, Oxford.

**Keywords:** cancer, clinical decision making, diagnosis, general practice, gut feeling, intuition

## Abstract

**Background:**

Growing evidence for the role of GPs’ gut feelings in cancer diagnosis raises questions about their origin and role in clinical practice.

**Aim:**

To explore the origins of GPs’ gut feelings for cancer, their use, and their diagnostic utility.

**Design and setting:**

Systematic review and meta-analysis of international research on GPs’ gut feelings in primary care.

**Method:**

Six databases were searched from inception to July 2019, and internet searches were conducted. A segregated method was used to analyse, then combine, quantitative and qualitative findings.

**Results:**

Twelve articles and four online resources were included that described varied conceptualisations of gut feelings. Gut feelings were often initially associated with patients being unwell, rather than with a suspicion of cancer, and were commonly experienced in response to symptoms and non-verbal cues. The pooled odds of a cancer diagnosis were four times higher when gut feelings were recorded (OR 4.24, 95% confidence interval = 2.26 to 7.94); they became more predictive of cancer as clinical experience and familiarity with the patient increased. Despite being included in some clinical guidelines, GPs had varying experiences of acting on gut feelings as some specialists questioned their diagnostic value. Consequently, some GPs ignored or omitted gut feelings from referral letters, or chose investigations that did not require specialist approval.

**Conclusion:**

GPs’ gut feelings for cancer were conceptualised as a rapid summing up of multiple verbal and non-verbal patient cues in the context of the GPs’ clinical knowledge and experience. Triggers of gut feelings not included in referral guidance deserve further investigation as predictors of cancer. Non-verbal cues that trigger gut feelings appear to be reliant on continuity of care and clinical experience; they tend to remain poorly recorded and are, therefore, inaccessible to researchers.

## INTRODUCTION

Clinician gut feeling is an acknowledged component of clinical decision making in primary care.^1^^–^^3^ In the clinical reasoning literature, the term ‘gut feeling’ is used interchangeably with ‘intuition’, ‘suspicion’, and ‘instinct’ making a precise conceptualisation elusive.^4^ Stolper’s definition of gut feeling describes *‘an uneasy feeling perceived by a GP as he/she is concerned about a possible adverse outcome, even though specific indications are lacking: There’s something wrong here.’* Additionally, Stolper’s definition includes a sense of reassurance, defined as *‘a secure feeling perceived by a GP about the further management and course of a patient’s problem, even though the doctor may not be certain about the diagnosis: Everything fits in.’* ^5^ This framing recognises that GPs often develop a clinical impression during the consultation that informs a diagnostic strategy rather than leading to a definitive diagnosis.^6^ However, gut feelings are regarded by some as overly subjective and prone to bias,^7^^,^^8^ and the product of ‘vanity’ or ‘paranoia’ that have the potential to cause harm to patients.^2^^,^^9^

The dual theory of cognition^10^^,^^11^ is long established as fast thinking (system one) encompassing heuristics, pattern recognition, and intuition, with slow thinking (system two) representing cognisant analytical or algorithmic approaches to decision making.^12^^–^^15^ Increasingly regarded as a false dichotomy, the cognitive continuum theory affords a middle ground, in which both systems are used to varying degrees.^16^ Gut feelings have been conceptualised as unconscious system-one processes that resist incorporation into guidelines;^1^^,^^17^^,^^18^ this puts them at odds with Western medical culture, which is dominated by analytical approaches epitomised by evidence-based guidelines in spite of there being evidence that slower analytical approaches do not necessarily lead to improved diagnostic decisions.^19^

GPs’ gut feelings have been reported to be more predictive of cancer than symptom combinations included in clinical guidelines,^20^ and a greater understanding of the basis of GP gut feelings may improve patient triage for cancer investigation.^21^ Gut feelings for cancer may have additional utility in primary care due to the relative lack of evidence and guidance for non-specific cancer presentations.^22^ The aim of this systematic review was to:

- examine the current evidence regarding GPs’ gut feelings for cancer;
- collate the factors that are thought to prompt the experience and use of gut feelings;
- explore how gut feelings are used in primary care; and
- establish the diagnostic utility of gut feelings through meta-analysis.

## METHOD

A systematic review was conducted in four stages:

- literature search;
- screening;
- quality appraisal; and
- data extraction and synthesis.^23^

**Table:** How this fits in

Clinician gut feelings are an acknowledged part of clinical decision making, but the literature on gut feelings lacks consistency. This systematic review found that GPs’ gut feelings may be predictive of cancer, and are conceptualised as a rapid summing up of multiple verbal and non-verbal patient cues in the context of the GPs’ knowledge and experience. Non-verbal cues that trigger gut feelings appear to be reliant on continuity of care and clinical experience, but remain poorly recorded and inaccessible to researchers. It is possible that gut feelings triggered by clinical features outside of cancer guidelines highlight the limitations of current referral criteria.

The review protocol was registered with PROSPERO (http://www.crd.york.ac.uk/PROSPERO/display_record.php?ID=CRD42018109001).

### Data sources and searches

In July 2019, a search for literature exploring GPs’ use of gut feelings for a potential cancer diagnosis was carried out in the following databases from inception: MEDLINE, Embase, PsycINFO, CINAHL, Web of Science, and Dissertations & Theses Global. A range of potential synonyms for ‘gut feeling’ were searched for, including ‘instinct’, ‘tacit knowledge’, and ‘intuition’, to ensure maximum capture of relevant publications. All search terms are detailed in Supplementary Table S1. Targeted internet searches were carried out in specific domains, for example, nhs.uk and org.uk websites, and those for the National Institute of Health and Care Excellence (NICE), Cancer Council Australia, Canadian Cancer Society, and Cancer Research UK (CRUK), as well as a broader Google Scholar search being undertaken. Internet search terms are outlined in Supplementary Table S2.

Reference lists of included articles were searched by hand by two researchers. When full-text articles were not accessible or the article abstract was from conference proceedings, the full text was requested from the relevant authors.

### Study selection

Published studies, theses, magazine articles, and websites describing gut feeling for the suspicion of cancer in primary care were included. Literature that described cancer suspicion that grew out of an uneasy feeling that was not necessarily based on clinical evidence was included, regardless of the terms used by authors. Studies were not excluded based on participant age, publication date, or language. The title, abstract, and full text of retrieved articles were independently screened by two reviewers; any disagreements were resolved through discussion with a third reviewer. Articles were included if they fulfilled the following criteria:

- population — clinicians working in primary care;
- focus — gut feelings;
- condition — cancer; and
- outcome — decision making, investigation, or eventual diagnosis.

### Data extraction and quality assessment

Study characteristics and findings were extracted into a pre-prepared Excel spreadsheet by two researchers independently; 20% of the studies were double extracted to check consistency.

The quality of studies was assessed by the researcher who carried out the data extraction. The quality of qualitative papers was assessed using the CASP Qualitative Study checklist.^24^ Quantitative studies were assessed using the Newcastle-Ottawa Scale adapted for cross-sectional studies^25^ or the CASP Cohort Study Checklist.^26^ The CASP Cohort Study Checklist was used instead of the Newcastle-Ottawa Scale for the assessment of cohort studies, as was originally planned, because more of the items in the CASP Checklist were applicable to the cohort studies included in this review. No studies were excluded based on quality assessment alone.

### Data synthesis and analysis

A segregated method was used to analyse quantitative and qualitative data separately before synthesising the findings.^27^ Qualitative data, including participant quotations and the secondary interpretations of study authors, were analysed using NVivo qualitative analysis software (version 12). An inductive thematic analysis was used to identify themes and subthemes.^28^

To analyse the quantitative data, common findings were identified across the studies. The pooled odds ratio (OR) for cancer diagnosis when the GP had reported gut feelings versus no gut feelings was derived using a random effects meta-analysis in RevMan (version 5.3). Finally, qualitative and quantitative data were combined into a descriptive overview of findings.

### Patient and public involvement

Five patients with cancer were involved in a workshop in November 2018 to discuss the interest and relevance to patients of GPs’ gut feelings about cancer, provide feedback on this study’s research questions, and advise on the dissemination of the results of this review.

## RESULTS

**Figure 1.**
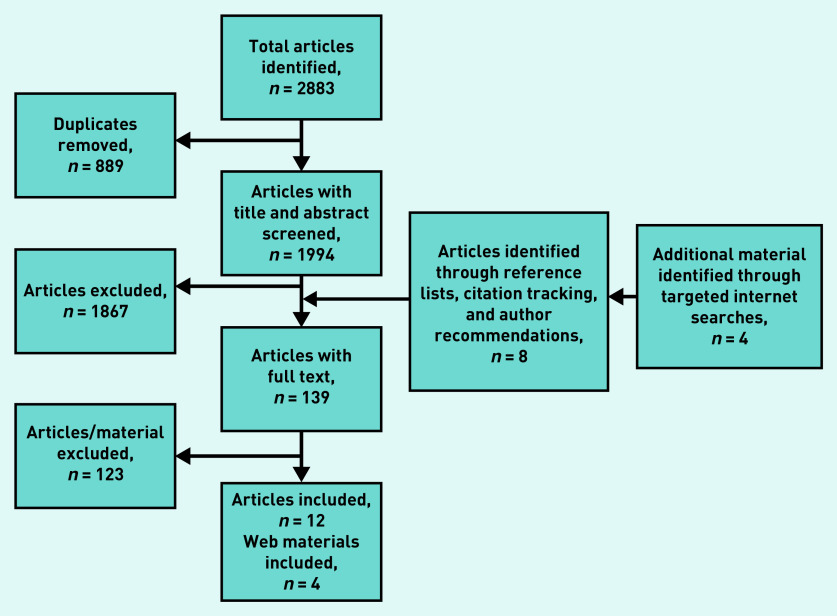
***PRISMA diagram of materials included in this review.***

Twelve articles were included, as outlined in the Preferred Reporting Items for Systematic Reviews and Meta-Analyses (PRISMA) flowchart (Figure 1). Four of the included articles originated from the UK,^29^^–^^32^ one was a multicentre European study,^33^ one stemmed from the Netherlands,^20^ three from Denmark,^34^^–^^36^ one was from Spain,^37^ and two were from Norway.^38^^,^^39^ The included articles comprised six qualitative studies,^29^^–^^32^^,^^37^^,^^39^ four prospective cohort studies,^20^^,^^33^^,^^34^^,^^38^ and two cross-sectional studies.^35^^,^^36^ Characteristics of the included studies are detailed in Table 1.

**Table 1.** Characteristics of included studies

**Study**	**Title**	**Country conducted**	**Study design**	**Participants, *n***	**Cancer type**
Bankhead (2005)^29^	Identifying potentially significant diagnostic factors for ovarian cancer in primary care: a qualitative and quantitative study	UK	Qualitative interviews	11 GPs, 40 patients	Ovarian cancer
Clarke *et al* (2014)^30^	‘Shouting from the roof tops’: a qualitative study of how children with leukaemia are diagnosed in primary care	UK	Qualitative interviews	21 parents, 9 GPs	Leukaemia
Donker *et al* (2016)^20^	Determinants of general practitioner’s cancer-related gut feelings — a prospective cohort study	The Netherlands	Prospective cohort	59 GPs	Cancer
Green *et al* (2015)^31^	Cancer detection in primary care: insights from general practitioners	UK	Qualitative interviews	55 GPs	Cancer
Hjertholm *et al* (2014)^34^	Predictive values of GPs’ suspicion of serious disease: a population-based follow-up study	Denmark	Prospective cohort	404 GPs	Cancer and serious disease
Holtedahl *et al* (2017)^33^	Abdominal symptoms in general practice: frequency, cancer suspicions raised, and actions taken by GPs in six European countries. Cohort study with prospective registration of cancer	Norway, Denmark, Sweden, Belgium, Netherlands, Scotland	Prospective cohort	493 GPs, 61 802 patients, 511 cancer patients	Abdominal cancer
Ingeman *et al* (2015)^35^	The Danish cancer pathway for patients with serious non-specific symptoms and signs of cancer — a cross-sectional study of patient characteristics and cancer probability	Denmark	Cross-sectional	1278 patients	Cancer
Johansen *et al* (2012)^39^	How does the thought of cancer arise in a general practice consultation? Interviews with GPs	Norway	Qualitative interviews	11 GPs	Cancer
Oliva *et al* (2016)^37^	Gut feelings in the diagnostic process of Spanish GPs: a focus group study	Spain	Qualitative focus groups	20 GPs	Cancer
Pedersen *et al* (2019)^36^	Patient–physician relationship and use of gut feeling in cancer diagnosis in primary care: a cross-sectional survey of patients and their general practitioners	Denmark	Cross-sectional	581 GPs 1200 patients	Cancer
Robinson (2016)^32^	What are the factors influencing GPs in the recognition and referral of suspected lung cancer?	UK	Qualitative interviews	36 GPs	Lung cancer
Scheel (2013)^38^	Cancer suspicion in general practice: the role of symptoms and patient characteristics, and their association with subsequent cancer	Norway	Prospective cohort	396 GPs, 51 073 patients, 261 patients with cancer	Cancer

Four additional materials were identified through internet searches: an NICE guideline,^40^ a report on the use of decision support tools,^41^ and one report and one website detailing innovative care pathways for cancer diagnosis.^42^^,^^43^

### Quality assessment

Details of the quality assessment can be found in Supplementary Table S3. Both the cohort and cross-sectional studies were regarded as using highly selected, unjustified samples, often with imbalances in GP age, experience, and sex. The qualitative papers generally fared well on the CASP Qualitative Studies Checklist and were considered to make a valuable contribution to the literature.

### Presence of gut feelings for cancer

Some GPs struggled to articulate what gut feelings were, even though they acknowledged their presence.^39^ As well as *‘gut feelings’*,^31^^,^^39^ a range of terms were used to describe gut feelings including *‘intuition’*,^39^ *‘alarm bells’*,^30^ *‘worry’*,^31^ and *‘suspicion’*.^34^ Gut feelings were sometimes described as a physical sensation, a *‘lurch of your stomach’*,^30^ or *‘hairs on the back of your neck’*;^32^ they were also often related to the patient being ‘unwell’ rather than being specific to a cancer diagnosis.^32^ This is consistent with Stolper’s ‘sense of alarm’ that occurs before the GP has an idea of what the diagnosis might be.^5^

### Contributors to gut feelings for cancer

Gut feelings were described as a rapid ‘summing up’ of multiple verbal and non-verbal patient cues in the context of GPs’ knowledge and experience.^20^^,^^30^^–^^32^^,^^39^ As Johansen *et al* (2012) stated:

*‘* [Gut feeling] *is the sum of all your knowledge, the sum of all your experience* … *all your knowing from reading updates, attending courses, all the patients you have had whom you* … *have investigated, referred and received feedback about. And then there is your knowledge of humankind and of the context, namely the person and patient and the community you work in.’* ^39^

Commonly, a GPs’ familiarity and knowledge of their patient allowed non-verbal cues to be noticed.^29^^,^^39^ In adults, non-verbal cues included inconsistencies with what was considered the patients ‘normal’ physical appearance^29^^,^^30^^,^^39^ or usual consulting frequency or behaviour, including the way patients sat or spoke during the consultation.^20^^,^^30^^,^^37^ This was noted in Bankhead’s (2005) study:

*‘Certainly if you know them well and if you see a dramatic change as they’re walking through the door, either weight loss or, there’s a colour about them often, they have a change in colour, it’s not a natural just anaemia, it’s just a magnolia colour often ... It’s just a gut instinct … I think you just have to know the patient.’* ^29^

Only one study reported that GPs were less likely to rely on gut feelings if they had good knowledge of their patient.^36^

Non-verbal cues in children included bad moods, irritability, or lack of enthusiasm.^30^ Such cues may well prove difficult to articulate or rationalise and, so, are consistent with Stolper’s definition.

Symptoms were the most common verbal cues. Specific symptoms, most often unintentional weight loss,^20^^,^^35^^,^^38^ prolonged symptom duration,^20^ and the presence of multiple symptoms,^38^ all contributed. However, Ingeman *et al* reported that when the patient presented with clear symptoms for cancer, gut feeling was rated as less important to the referral by the GPs.^35^

GPs postulated an association between clinical experience and the accuracy of gut feelings through pattern recognition, built up through the repetition of clinical scenarios, suggesting that their ‘diagnostic memory’ improved the accuracy of their gut feelings.^30^^,^^32^ As Robinson noted:

*‘I think intuition isn’t quite the pseudo thing people think it is, I think it’s actually, a combination of having seen lots and lots and lots of cases and you’ve seen lots of scenarios and, and it’s all accumulated in, in your subconscious mind, as well as your conscious mind and, and you, you’re actively drawing on this.’* ^32^

In one study, 43% of patients who were referred based on a gut feeling of GPs with >15 years’ experience received a cancer diagnosis within 3 months, compared with 26% of patients referred by GPs with <15 years’ experience.^20^ Furthermore, for every year increase in the GP’s age, the positive predictive value (PPV) of gut feelings for cancer increased by 3%.^20^ In addition, GPs who rated their empathy levels highly were more likely to report using gut feelings;^36^ however, some warned that overconfidence in gut feelings could lead to missed cues or reaching the wrong conclusion, especially in GPs who were less experienced.^32^

### Actions prompted by a gut feeling

Gut feelings served as a prompt to re-examine the patient’s narrative,^30^ to think beyond the most likely clinical explanation for symptoms,^30^^,^^39^ and to request further testing or specialist referral.^20^^,^^30^ In one Norwegian prospective cohort study, 89.5% of patients were investigated when GPs had a gut feeling for cancer compared with 30.6% when there was no gut feeling.^38^ In a Danish study, survey responses showed that the presence of gut feelings resulted in increased referrals (prevalence ratio 2.56, 95% confidence interval [CI] = 2.22 to 2.96) and increased the likelihood of a scheduled follow-up appointment (prevalence ratio 1.15, 95% CI = 1.05 to 1.26).^34^

When not referring, GPs reported that they managed gut feelings through *‘watchful waiting’* or *‘safety netting’* by encouraging patients to return if their symptoms did not improve.^20^^,^^30^ Gut feelings, in the form of a sense of reassurance, were rarely mentioned; when they were, they were described as prompting the watchful-waiting approach, which allowed further tests to be delayed, particularly when further patient contact was possible as a *‘safety measure’*.^37^

### Diagnostic value of gut feelings in identifying cancer

All studies that explored the diagnostic value of gut feelings in the identification of cancer reported a diagnostic utility. In a prospective cohort study, the odds for a subsequent new diagnosis of cancer was 2.11 (95% CI = 1.15 to 3.89), and 8.89 (95% CI = 1.49 to 53.02) for a recurrent cancer when a gut feeling was recorded.^33^ A Danish study reported that the PPV of GPs’ gut feelings for cancer or other serious disease was 9.8 (95% CI = 6.4 to 14.1) within 2 months of the initial consultation, and 16.4 (95% CI = 12.1 to 21.5) up to 6 months after the initial consultation.^34^ GPs were six times more likely to have suspected cancer than not to have suspected cancer, if cancer was subsequently diagnosed.^38^

Meta-analysis of the four studies^33^^–^^35^^,^^38^ reporting a cancer conversion rate for when gut feeling was recorded by a GP showed that the odds of the patient being diagnosed with cancer were four times higher than when no gut feeling was recorded (OR 4.24, 95% CI = 2.26 to 7.94) (Table 2). The level of heterogeneity was high (I^2^ = 87%). Excluding the outlying study, Ingeman *et al,*^35^ removed the heterogeneity (I^2^ = 0%) and increased the OR (5.43, 95% CI = 4.15 to 7.09) (data not shown).

**Table 2.** Odds of cancer diagnosis with gut feeling

**Study**	**Gut feeling, *n***		**No gut feeling, *n***		**Weight, %**	**Odds ratio, M-H random (95% CI)**	
	**Cancer diagnoses**	**Total patients**	**Cancer diagnoses**	**Total patients**			
Hjertholm *et al* (2014)^34^	8	256	22	4262	19.8	6.22 (2.74 to 14.11)	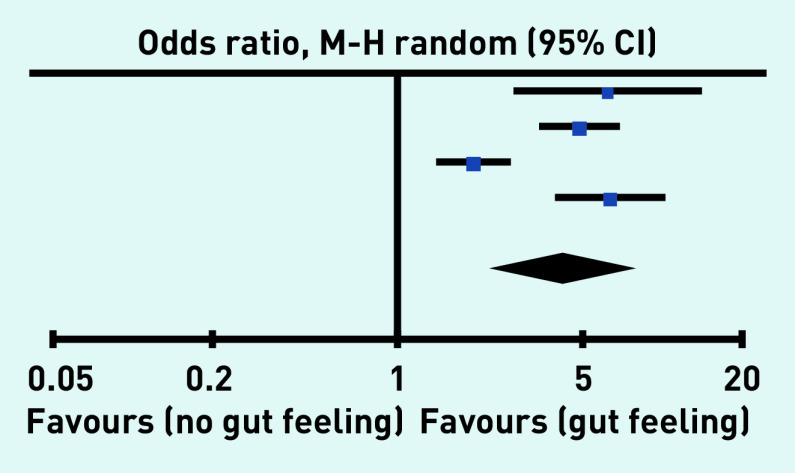
Holtedahl *et al* (2017)^33^	64	1097	65	5167	27.3	4.86 (3.42 to 6.91)	
Ingeman *et al* (2015)^35^	69	287	138	982	27.6	1.94 (1.40 to 2.68)	
Scheel (2014)^38^	58	1515	24	3854	25.4	6.35 (3.93 to 10.26)	
**Total**	**—**	**3155**	**—**	**14 265**	**100**	**4.24 (2.26 to 7.94)**	
**Total cancer diagnoses**	**199**	**—**	**249**	**—**	**—**	**—**	

Heterogeneity: Tau^2^= 0.34; c^2^= 23.96; degrees of freedom = 3 (P < 0.0001); l^2^ = 87%. Test for overall effect: Z = 4.51 (P < 0.00001). M-H = Mantel-Haenszel.

### Gut feelings for cancer in policy and practice

Gut feelings were included in some UK cancer referral guidelines and policy documents: the NICE guideline on suspected cancer states that GPs should trust their own and their patients’ intuition, but includes no recommendations about how to apply this in practice.^40^ A report by NHS England, Cancer Research UK, and Macmillan Cancer Support suggested that GPs could use cancer risk-prediction tools to help *‘legitimise’* fast-track referrals based on gut feelings,^41^ although this approach would depend on gut feelings being triggered by known symptoms or risk factors.

Cancer pathways that include GPs’ gut feelings as an explicit referral criterion, either alone or in combination with other symptoms, are being trialled in Europe.^35^^,^^42^^,^^43^ Gut feelings were the second most common reason for referral to the Danish pathway for non-specific cancer symptoms, and cancer was more likely to be diagnosed when gut feeling was a strong influence on the GP’s decision to refer (prevalence ratio 2.57, 95% CI = 1.31 to 5.05).^35^

GPs reported varying success of integrating gut feelings into clinical practice: some were able to refer patients based on gut feeling,^20^^,^^30^ but others recounted instances when referrals made because of a gut feeling had been rejected by specialist colleagues due to a perceived lack of clinical evidence.^39^ These patients were later diagnosed with cancer, suggesting that earlier diagnosis may have been possible. GPs also reported leaving their gut feelings out of referral decisions due to their own perception that they were only based on a feeling and *‘nothing big’*.^39^

The place of gut feelings in evidence-based medicine was discussed in two qualitative studies of British GPs: in these studies, GPs generally felt that gut feelings were complementary to analytical reasoning and were of particular benefit when the patient’s symptoms did not fit guidelines:

*‘In general practice, there’s always room for that kind of, well gut feeling … You know, you can only take those things* [guidelines; risk scores] *to a certain level, but you’ve kind of got to use your common sense and experience and your kind of, I’m just worried about this patient, you know, I need to do something here.’* (Green *et al*)^31^

*‘I think you should have both* [analytical and intuitive reasoning]*, both working together. I don’t think you should dismiss the intuitive, I think the intuitive is always going to be an important part of general practice, which is actually an art. Intuitively you are analysing, it’s just in a different type of way.’*

*(Robinson)^32^*

Some GPs, however, expressed concerns that over-reliance on gut feelings meant that alternative hypotheses would not be considered, potentially leading to diagnostic error.^32^

## DISCUSSION

### Summary

GPs conceptualised gut feeling as an uneasy feeling, typically triggered by a rapid summing up of multiple verbal and non-verbal cues. A cancer diagnosis was more likely in patients for whom the GP had a gut feeling compared with patients for whom the GP experienced no gut feeling. Gut feelings for cancer were often prompted by patterns of clinical features — commonly, symptoms — and were reported to be based on deviations from patients’ usual presentation or behaviour. They were linked with GPs’ empathy, and developed with experience or through repetition of clinical scenarios. Gut feelings were considered complementary to evidence-based practice, being included in some clinical guidelines and as entry criteria to cancer investigation. GPs described varying experiences of incorporating their gut feelings into specialist referrals.

### Strengths and limitations

By using a broad search strategy that included varied terms to describe gut feeling, and by including theses, internet sites, and published research, the likelihood that all relevant literature would be identified was increased. By employing established segregated methods to synthesise the literature descriptively, thematically, and, where possible, through meta-analysis, the authors are likely to have developed the most detailed and representative interpretation of the current evidence base for gut feelings for cancer in primary care.

This study does, however, have a number of limitations. Despite the inclusive approach, only 16 relevant data sources were identified. The literature was limited to studies that report experiences of gut feelings, suggest definitions of gut feelings, or document the circumstances leading to gut feelings for cancer in primary care. There remained great variation in the terminology used and no objective measure of ‘gut feelings’, despite attempts by the research community to standardise a definition. In the meta-analysis, a random-effects model was used with, and without, outlying studies to account for the high levels of heterogeneity.^44^ Heterogeneity reduced to zero when one outlying study was removed. This prospective cohort study used the term ‘gut feeling’, whereas the other cross-sectional studies conceptualised gut feeling as ‘suspicion’ or ‘intuition’. These differences highlight that greater consistency is needed in the conceptualisation of gut feeling during both study conduct and reporting.

The literature was also entirely limited to European studies. This is, perhaps, unsurprising, given the existence of dedicated European research groups, such as the Cogita Network (http://www.gutfeelings.eu), that have set specific agendas to study gut feeling in European primary care. However, it does raise questions about whether gut feelings are conceptualised outside of European primary care practice, and also, therefore, about the generalisability of the findings to non-European settings.

Lastly, there were imbalances in the study populations in relation to age, sex, and years of experience — characteristics that are cited as contributory to the development, use, and accuracy of gut feelings. An over-representation of older, more-experienced GPs could make gut feelings seem more reliable. In the qualitative studies, purposive sampling was used with the intention of recruiting diverse samples based on characteristics such as sex and years of clinical experience, but as these do not take into account personal (for example, empathy) or cultural factors that could influence use of gut feelings, important perspectives may remain under represented.

### Comparison with existing literature

The most widely cited definition of GP gut feeling describes a sense of reassurance or alarm that may occur in the absence of symptoms.^2^^,^^45^ However, gut feelings for cancer in the absence of symptoms were uncommon in this review, except in a small number of qualitative descriptions of past clinical encounters; this is not the case across disease areas as qualitative and quantitative studies of acute childhood illness, pulmonary embolism, and acute coronary syndrome have reported gut feelings unrelated to presenting symptoms.^46^^–^^48^ In these studies, triggers for gut feeling are described as a patient’s appearance not being a *‘typical coronary heart disease patient picture’* ^48^ or there being a *‘sudden change in the patient’s condition’*.^46^ Van den Bruel *et al* reported that the presence of gut feelings significantly increased the likelihood that a child had a serious illness when the clinical presentation pointed to a non-serious infection, regardless of the child’s age or the eventual diagnosis.^47^

The OR from the reviewers’ meta-analysis (OR 4.24, 95% CI = 2.26 to 7.94) supports previous research that the presence of a gut feeling could be used as an indicator of serious illness.^47^ In comparison, the ORs from case–control studies using primary care records data for cancer symptoms ranged from 2.7 to 86 for lung cancer,^49^ from 1.4 to 15 for pancreatic cancer,^50^ from 2.1 to 20 for colorectal cancer,^51^ and from 1.3 to 11.4 for myeloma.^52^ The individual ORs for nausea and vomiting for pancreatic cancer (OR 4.5, 95% CI = 3.5 to 5.7),^50^ weight loss (OR 4.3, 95% CI = 2.2 to 8.2),^49^ and dyspnoea (OR 4.7, 95% CI = 2.7 to 8.0)^49^ in lung cancer were similar to the ORs for gut feeling in this review.

The authors found little mention of a sense of reassurance when GPs reported their own experiences of gut feelings for cancer.^53^ Qualitative study participants were usually free to choose their own definitions, rather than use a standardised terminology provided by researchers. GPs might be reluctant to volunteer taking no further action based on a reassuring gut feeling in the face of symptoms of cancer. The sense of reassurance has primarily been conceptualised during Delphi exercises, in which GPs were asked to reflect on the nature of gut feelings;^5^^,^^54^ where mentioned, GPs were more cautious about relying on a sense of reassurance than acting on a sense of alarm.^37^ Stolper *et al* suggested that a failure to develop a sense of alarm may be described as an inappropriate sense of reassurance, but it remains unclear whether an absence of alarm equates to a sense of reassurance.^55^ Research focusing on the detection of serious disease may have neglected the sense of reassurance, it may not be experienced in the context of a possible cancer diagnosis, or it may be less of a feature of clinical practice than previously suggested.

Gut feelings have now been reported in qualitative and quantitative studies across primary and secondary care.^3^^,^^56^ Analytical and non-analytical reasoning are considered complementary in the theoretical literature.^16^^,^^18^ However, physician narratives confirm a resistance to GP gut feeling as a valid referral criterion due to the apparent incompatibility of gut feelings with evidence-based practice.^9^ In response, GPs have reported ignoring their gut feelings^9^^,^^57^^,^^58^ or choosing investigations for which specialist triage is not involved.^53^

### Implications for research and practice

A major motivation for this review was to examine whether, and how, gut feelings could be integrated into clinical guidance for suspected cancer in primary care. Current guidelines for suspected cancer base referral recommendations on patient characteristics, risk factors, symptoms, signs, and test results.^40^ Gut feelings based on these clinical features can be, and are, incorporated into clinical guidelines if there is an evidence base to support the feature in question. The unanswered question is whether the presence of a gut feeling, in these cases, represents anything more than the recognition of an ‘alarm’ feature.

It is possible that research into gut feelings captures combinations of clinical features that fall outside of existing cancer referral guidelines, highlighting limitations of the current evidence base; for example, gut feelings were particularly noted in response to non-specific symptoms and in patients with multiple symptoms. Future research should focus on scenarios when gut feeling is triggered by combinations of patient characteristics, risk factors, and clinical features that are not included in current guidelines, especially if subsequent investigation leads to a cancer diagnosis.

Multivariable approaches to cancer risk prediction may better capture the complex associations that are present in clinical practice,^59^ but uptake into routine primary care practice is poor^60^ and models are built using coded electronic health records data,^61^ thereby excluding free-text data that is more likely to contain information recorded about non-verbal cues.^62^ Non-verbal cues, such as deviations from patients’ usual presentation pattern or appearance, represent aspects of clinical practice that are more difficult to standardise and codify, making their integration into evidence-based guidelines problematic. Processes for documenting and retrieving gut feelings and non-verbal cues should be established, given that this is a known barrier to gut feelings being used in clinical decision making.^9^ This may pave the way for the acceptance of gut feelings by specialists and guideline developers, thereby following the lead of NICE.^40^

For non-verbal cues to be predictive of cancer, it is clear from this review that a knowledge and understanding of the patient’s ‘normal’ must be built over time.^37^ Within fragmented and overstretched primary care systems, there is a danger that the relational and empathic aspects of the GP–patient relationship, which are repeatedly cited as contributors to gut feeling, could be damaged. Ensuring relational continuity of care is at odds with the increasing workload, fragmentation, part-time working, and shorter consultations observed in modern-day primary care that, paradoxically, necessitates faster, non-analytic approaches to decision making.^63^^,^^64^

To the authors’ knowledge, only Stolper *et al* ^2^ have explored whether gut feelings can be taught to GP trainees. During focus groups, GPs reported that gut feelings could be taught by encouraging reflection on their diagnostic reasoning, and paying attention to both how patients present themselves and their illness, and the reaction that this provokes in the trainee.^2^ Research is required to test methods for educating GP trainees in the recognition or use of gut feelings, and whether education would improve actions following a gut feeling.^4^^,^^65^ Van den Bruel *et al* suggested three actions following a gut feeling: careful clinical examination; seeking input from colleagues, by referral if necessary; and provision of carefully worded advice or safety netting.^47^ The authors agree with Van den Bruel *et al* but suggest that research is needed to establish how gut feelings can be effectively communicated to colleagues and patients.

The origins of gut feelings in clinical practice have been debated for decades; this, together with the recent upsurge in interest in GPs’ gut feelings and the gaps in understanding that have been highlighted by this review, indicate that the debate is far from over. Gut feelings are reported in response to patterns of clinical features that could be included in clinical guidance. These triggers deserve dedicated investigation as predictors of cancer, especially if not already included in referral guidelines.

## References

[1] Lee J, Chan ACM, Phillips DR (2006). Diagnostic practise in nursing: a critical review of the literature. Nurs Health Sci.

[2] Stolper E, van Bokhoven M, Houben P (2009). The diagnostic role of gut feelings in general practice. A focus group study of the concept and its determinants.. BMC Fam Pract.

[3] Van den Brink N, Holbrechts B, Brand PLP (2019). Role of intuitive knowledge in the diagnostic reasoning of hospital specialists: a focus group study. BMJ Open.

[4] Stolper E, Van de Wiel M, Van Royen P (2011). Gut feelings as a third track in general practitioners’ diagnostic reasoning. J Gen Intern Med.

[5] Stolper E, Van Royen P, Van de Wiel M (2009). Consensus on gut feelings in general practice. BMC Fam Pract.

[6] Summerton N (2011). Primary care diagnostics: the patient-centred approach in the new commissioning environment.

[7] Guyatt G, Cairns J, Churchill D (1992). Evidence-based medicine: a new approach to teaching the practice of medicine. JAMA.

[8] Sackett DL, Rosenberg WMC, Muir Gray JA (1996). Evidence based medicine: what it is and what it isn’t. BMJ.

[9] Peters A, Vanstone M, Monteiro S (2017). Examining the influence of context and professional culture on clinical reasoning through rhetorical-narrative analysis. Qual Health Res.

[10] Ferreira MB, Garcia-Marques L, Sherman SJ, Sherman JW (2006). Automatic and controlled components of judgment and decision making. J Pers Soc Psychol.

[11] Kahneman D (2011). Thinking, fast and slow.

[12] Velanovich V (1994). Bayesian analysis in the diagnostic process. Am J Med Qual.

[13] Eva KW (2005). What every teacher needs to know about clinical reasoning. Med Educ.

[14] O’Neill ES (1995). Heuristics reasoning in diagnostic judgment. J Prof Nurs.

[15] Marewski JN, Gigerenzer G (2012). Heuristic decision making in medicine. Dialogues Clin Neurosci.

[16] Custers EJFM (2013). Medical education and cognitive continuum theory: an alternative perspective on medical problem solving and clinical reasoning. Acad Med.

[17] Rew L, Barrow EM (2007). State of the science: intuition in nursing, a generation of studying the phenomenon. ANS Adv Nurs Sci.

[18] Greenhalgh T (2002). Intuition and evidence — uneasy bedfellows?. Br J Gen Pract.

[19] Norman G, Sherbino J, Dore K (2014). The etiology of diagnostic errors: a controlled trial of system 1 versus system 2 reasoning. Acad Med.

[20] Donker GA, Wiersma E, van der Hoek L, Heins M (2016). Determinants of general practitioner’s cancer-related gut feelings — a prospective cohort study. BMJ Open.

[21] Hamilton W (2009). Five misconceptions in cancer diagnosis. Br J Gen Pract.

[22] Buntinx F, Mant D, Van den Bruel A (2011). Dealing with low-incidence serious diseases in general practice. Br J Gen Pract.

[23] Mahtani KR, Jefferson T, Heneghan C (2018). What is a ‘complex systematic review’? Criteria, definition, and examples. BMJ Evid Based Med.

[24] (2018). Critical Appraisal Skills Programme. CASP Qualitative Checklist 2018.

[25] Modesti PA, Reboldi G, Cappuccio FP (2016). Panethnic differences in blood pressure in europe: a systematic review and meta-analysis. PloS One.

[26] (2018). Critical Appraisal Skills Programme. CASP Cohort Study Checklist 2018.

[27] The Joanna Briggs Institute (2014). The Joanna Briggs Institute Reviewers’ Manual 2014: the systematic review of economic evaluation evidence.

[28] Boyatzis RE (1998). Transforming qualitative information: thematic analysis and code development.

[29] Bankhead CR (2005). Identifying potentially significant diagnostic factors for ovarian cancer in primary care: a qualitative and quantitative study.

[30] Clarke RT, Jones CH, Mitchell CD, Thompson MJ (2014). ‘Shouting from the roof tops’: a qualitative study of how children with leukaemia are diagnosed in primary care. BMJ Open.

[31] Green T, Atkin K, Macleod U (2015). Cancer detection in primary care: insights from general practitioners. Br J Cancer.

[32] Robinson S What are the factors influencing GPs in the recognition and referral of suspected lung cancer?.

[33] Holtedahl K, Vedsted P, Borgquist L (2017). Abdominal symptoms in general practice: frequency, cancer suspicions raised, and actions taken by GPs in six European countries. Cohort study with prospective registration of cancer. Heliyon.

[34] Hjertholm P, Moth G, Ingeman ML, Vedsted P (2014). Predictive values of GPs’ suspicion of serious disease: a population-based follow-up study. Br J Gen Pract.

[35] Ingeman ML, Christensen MB, Bro F (2015). The Danish cancer pathway for patients with serious non-specific symptoms and signs of cancer — a cross-sectional study of patient characteristics and cancer probability. BMC Cancer.

[36] Pedersen AF, Andersen CM, Ingeman ML, Vedsted P (2019). Patient–physician relationship and use of gut feeling in cancer diagnosis in primary care: a cross-sectional survey of patients and their general practitioners. BMJ Open.

[37] Oliva B, March S, Gadea C (2016). Gut feelings in the diagnostic process of Spanish GPs: a focus group study. BMJ Open.

[38] Scheel BI (2013). Cancer suspicion in general practice: the role of symptoms and patient characteristics, and their association with subsequent cancer. Br J Gen Pract.

[39] Johansen ML, Holtedahl KA, Rudebeck CE (2012). How does the thought of cancer arise in a general practice consultation? Interviews with GPs. Scand J Prim Health Care.

[40] National Collaborating Centre for Cancer Suspected cancer: recognition and referral NICE guideline Full guideline. June 2015.

[41] Robinson S, Poirier V, Watson S (2017). Using Cancer Decision Support Tools to support the early diagnosis of cancer.

[42] Public Health Wales (2017). Innovative new clinic helps faster cancer diagnosis. http://www.wales.nhs.uk/sitesplus/888/news/45820.

[43] Accelerate, Coordinate, Evaluate (ACE) Programme (2018). Multidisciplinary Diagnostic Centre (MDC) based pathways for patients with non-specific but concerning symptoms: interim report.

[44] Higgins JPT, Green S (2011). Cochrane handbook for systematic reviews of interventions. Version 5.1.0.

[45] Stolper E, van Royen P, Dinant GJ (2010). The ‘sense of alarm’ (‘gut feeling’) in clinical practice. A survey among European general practitioners on recognition and expression. Eur J Gen Pract.

[46] Barais M, Morio N, Cuzon Breton A (2014). “I can’t find anything wrong: it must be a pulmonary embolism”: diagnosing suspected pulmonary embolism in primary care, a qualitative study. PLoS One.

[47] Van den Bruel A, Thompson M, Buntinx F, Mant D (2012). Clinicians’ gut feeling about serious infections in children: observational study. BMJ.

[48] Bruyninckx R, Van den Bruel A, Hannes K (2009). GPs’ reasons for referral of patients with chest pain: a qualitative study. BMC Fam Pract.

[49] Hamilton W, Peters TJ, Round A, Sharp D (2005). What are the clinical features of lung cancer before the diagnosis is made? A population based case-control study. Thorax.

[50] Stapley S, Peters TJ, Neal RD (2012). The risk of pancreatic cancer in symptomatic patients in primary care: a large case–control study using electronic records. Br J Cancer.

[51] Hamilton W, Lancashire R, Sharp D (2009). The risk of colorectal cancer with symptoms at different ages and between the sexes: a case–control study. BMC Med.

[52] Shephard EA, Neal RD, Rose P (2015). Quantifying the risk of multiple myeloma from symptoms reported in primary care patients: a large case–control study using electronic records. Br J Gen Pract.

[53] Vanstone M, Monteiro S, Colvin E (2019). Experienced physician descriptions of intuition in clinical reasoning: a typology. Diagnosis (Berl).

[54] Le Reste J-Y, Coppens M, Barais M (2013). The transculturality of ‘gut feelings’. Results from a French Delphi consensus survey. Eur J Gen Pract.

[55] Stolper E, Legemaate J, Dinant GJ (2010). How do disciplinary tribunals evaluate the “gut feelings” of doctors? An analysis of Dutch tribunal decisions, 2000–2008. J Law Med.

[56] Iqbal IZ, Kara N, Hartley C (2015). Gut instinct: a diagnostic tool?. J Laryngol Otol.

[57] Woolley A, Kostopoulou O (2013). Clinical intuition in family medicine: more than first impressions. Ann Fam Med.

[58] Goyder CR, Jones CHD, Heneghan CJ, Thompson MJ (2015). Missed opportunities for diagnosis: lessons learned from diagnostic errors in primary care. Br J Gen Pract.

[59] Usher-Smith J, Emery J, Hamilton W (2015). Risk prediction tools for cancer in primary care. Br J Cancer.

[60] Price S, Spencer A, Medina-Lara A, Hamilton W (2019). Availability and use of cancer decision-support tools: a cross-sectional survey of UK primary care. Br J Gen Pract.

[61] Hippisley-Cox J, Coupland C (2015). Development and validation of risk prediction algorithms to estimate future risk of common cancers in men and women: prospective cohort study. BMJ Open.

[62] Price SJ, Stapley SA, Shephard E (2016). Is omission of free text records a possible source of data loss and bias in Clinical Practice Research Datalink studies? A case–control study. BMJ Open.

[63] Hobbs FDR, Bankhead C, Mukhtar T (2016). Clinical workload in UK primary care: a retrospective analysis of 100 million consultations in England, 2007–14. Lancet.

[64] Round T, Steed L, Shankleman J (2013). Primary care delays in diagnosing cancer: what is causing them and what can we do about them?. J R Soc Med.

[65] Stolper CF, Van de Wiel MWJ, Hendriks RHM (2015). How do gut feelings feature in tutorial dialogues on diagnostic reasoning in GP traineeship?. Adv Health Sci Edu Theory Pract.

